# The economics of early inequality

**DOI:** 10.1098/rstb.2022.0293

**Published:** 2023-08-14

**Authors:** Gregory K. Dow, Clyde G. Reed

**Affiliations:** Department of Economics, Simon Fraser University, Burnaby, British Columbia, Canada V5A 1S6

**Keywords:** economic prehistory, Malthus, property rights, insider–outsider inequality, elite–commoner inequality, stratification

## Abstract

We examine three recent frameworks that attempt to explain early inequality. One explanation involves the emergence of dense and predictable resource patches in the Holocene, together with differential asset accumulation and inheritance by individuals or households. In this view, agriculture and pastoralism led to greater inequality because farmland and animal herds were readily inherited. Another explanation involves the distinction between ideal free and ideal despotic population distributions, together with factors that could trigger a transition from the former to the latter. We offer a third framework based on economic concepts. In our view, inequality initially arose across locations (*insider–outsider inequality*) and reflected geographical differences in resource endowments at those locations. As population densities increased, the barriers to individual migration across locations included fewer kinship linkages and the use of force by insiders to exclude outsiders. These barriers became important with the transition from mobile to sedentary foraging and predate agriculture. Insider–outsider inequality was followed by stratification within settlements (*elite–commoner inequality*), which arose at still higher population densities. We see these three theoretical approaches as distinct but complementary. While they overlap, each emphasizes some phenomena and processes ignored by the other two.

This article is part of the theme issue ‘Evolutionary ecology of inequality’.

## 1. Introduction

There is broad agreement that 15 000 years before the present (BP), almost all humans lived in small mobile foraging bands. By 5000 BP, the first states had arisen in Mesopotamia and Egypt. The intervening 10 000 years saw a transition from egalitarian societies to stratification involving elite and commoner classes, where the elites controlled access to land, inherited their privileges and often enjoyed vastly better living standards than commoners.

Contemporary mobile foragers tend to operate in bands of a few dozen people who make seasonal rounds within a traditional territory. Band sizes vary with ecological and technological conditions [1]. Social norms favour food sharing and oppose self-aggrandizement (see [2, pp. 46–51], and sources cited there). Anthropologists have offered several reasons for the prevalence of egalitarianism among mobile foragers: (i) the production technology is simple; (ii) natural resources are available to everyone; (iii) personal asset accumulation is limited; (iv) food storage is limited; (v) technology for violence is widely accessible; (vi) teamwork can be useful for hunting; and (vii) food sharing mitigates individual risks associated with bad luck or injuries. Prehistoric mobile foragers probably had similar characteristics and thus would also have been highly egalitarian.

Sedentary foraging became important in southwest Asia during 15 000–13 000 BP and developed in many other regions in the early Holocene. We have examined the reasons for this transition elsewhere ([3;4, ch. 4]). Ethnography establishes that sedentary foragers generally have much larger group sizes than mobile foragers, higher population relative to natural productivity, more food storage and more stratification [5–7, pp. 40–44;^1^, pp. 171–172]. Kelly [1, p. 104] remarks that such societies tend to exhibit ‘social hierarchies and hereditary leadership, political dominance, gender inequality, and unequal access to resources’, although these features are not universal.

Pristine transitions to agriculture occurred in 8–10 regions of the world, with the earliest of these dating roughly to the Pleistocene–Holocene climate boundary around 11 600 BP [4, ch. 5;^8^]. Agricultural productivity gradually rose through learning by doing and domestication. Such societies had much larger regional populations and settlement sizes than sedentary foragers [9]. Inequality was relatively modest for early labour-limited farming economies, but it increased dramatically as these economies became more land-limited [10].

Chapter 6 of our recent book on economic prehistory [4] presents a detailed formal model showing how early inequality could have emerged. One goal here is to describe this model in a verbal way that will be accessible to non-economists. The other main goal is to compare our theoretical framework with two other frameworks in the literature. We regard the three approaches as complementary. Although there are some areas of overlap, each approach helps to account for phenomena that are ignored or downplayed by the other two.

Section 2 describes an archaeological synthesis that centres on the Holocene climate, the emergence of concentrated and reliable resource patches, and the transmission of material assets like land and animal herds from parents to children. For convenience, we call this the ‘Holocene environment household inheritance’ theory, or HEHI. Section 3 summarizes a different theory, which focuses on the distribution of a population across a region, with varying assumptions about the ability of early arrivers to exclude late arrivers. We call this the ‘ideal free ideal despotic’ theory, or IFID.

Section 4 describes our framework, which we call the ‘insider outsider elite commoner’ theory, or IOEC. In our causal system, climate, geography and technology determine aggregate regional population through long-run Malthusian dynamics. In the short run, migration among individual sites determines local populations, local property rights and an associated pattern of inequality. Section 5 adds some caveats involving kinship and warfare. Section 6 compares our IOEC theory with the HEHI and IFID theories from §§2 and 3.

Section 7 is concerned with empirical issues. We briefly describe recent archaeological research on the origins of inequality in several regions. These findings offer partial support for our IOEC theory. Section 8 offers concluding thoughts.

## 2. The Holocene environment and household inheritance

The HEHI theory of early inequality originated with Borgerhoff Mulder *et al*. [11], who distinguished three kinds of wealth (embodied, material and relational) that can be passed from parents to children. Embodied wealth refers to individual characteristics like body weight or grip strength, material wealth refers to physical assets like land or cattle, and relational wealth refers to social assets like the number of one's exchange partners. When parents reliably transmit such wealth to their children, random shocks to the wealth levels of households in one generation yield persistent inequality across households in subsequent generations.

These authors define four economic systems: hunter–gatherer, horticultural, agricultural and pastoral. Hunter–gatherers use wild plant and animal foods, while the other three use domesticated plants or animals. Horticultural societies differ from agricultural societies in three ways: they are labour-limited rather than land-limited, they do not have land markets, and they do not use ploughs. Agriculturalists rely mainly on plants while pastoralists rely mainly on animals.

The four economic systems and the three wealth types yield 12 possible combinations [11, p. 685]. For each combination, the authors give an estimate of the relative importance of a specific wealth type in a specific economic system based on the opinions of expert ethnographers for 21 small-scale societies. They also estimate the heritability of each wealth type in each economic system based on regression results for parent–child pairs in the same societies. Weighting the wealth types by their relative importance provides an aggregate heritability estimate for overall wealth in each economic system. A similar weighting exercise generates an aggregate Gini coefficient for inequality within each economic system. The main findings are that: (i) material wealth is especially important in agricultural and pastoral societies; (ii) material wealth is more readily inherited than embodied or relational wealth in agricultural and pastoral societies; and (iii) both agriculturalists and pastoralists have substantially greater inequality than hunter–gatherers or horticulturalists (with the latter two tending to be similar).

This approach is folded into a larger synthesis by Mattison *et al*. [12] and Smith *et al*. [13], who stress three preconditions for persistent institutionalized inequality: climate stability, economic defensibility and intergenerational wealth transmission. During the Pleistocene, large and frequent climate shocks favoured high mobility and encouraged risk mitigation through norms of sharing. The transition from the Pleistocene to the Holocene led to a more stable climate and sometimes provided dense, reliable and spatially concentrated resource patches. These patches facilitated sedentism and were often worth defending. ‘[T]he ability to defend resources likely depend[ed] not only on steep resource gradients but also on group size’ [13, p. 12], where greater sedentism tended to promote larger group sizes. These factors led to enhanced roles for material wealth, individual property rights and differential wealth accumulation. In some regions, such developments were accompanied by plant and animal domestication. The result was intergenerational wealth transmission and persistent institutionalized inequality, as in the framework of Borgerhoff Mulder *et al*. [11].

## 3. Ideal free and ideal despotic distributions

The IFID framework for investigating inequality in small-scale societies focuses on the distribution of a regional population across sites or habitats. The central concepts are ‘ideal free distributions’ (IFDs) and ‘ideal despotic distributions’ (IDDs). Early work by archaeologists included Kennett *et al*. [14], Kennett & Winterhalder [15], Kennett *et al*. [16] and Shennan [17,18]. For general discussions, see Codding & Bird [19] and Weitzel & Codding [20].

In such models, each agent seeks a site with maximum *suitability*, which is defined to be biological fitness or a closely related variable like food intake. Suitability does not depend solely on the environmental features of a site such as elevation, watershed size or soil quality. Holding natural resources and technology constant, suitability also depends on the number of agents using the site, and it declines (at least eventually) as more agents arrive.

For an IFD, any agent can use any site, and the agents at a site all achieve the same suitability. Equilibrium requires that no agent wants to change sites. Thus, the regional population must be distributed so that all occupied sites have equal suitability, while the unoccupied sites have lower suitability levels. The outcome is therefore egalitarian both across and within sites.

For an IDD, early arrivers can defend claims to the best sites, and their individual suitability levels are not reduced by later arrivers who occupy less desirable sites. Social barriers to entry at the most valuable sites allow agents to achieve higher suitability than they would have at IFD population levels. Conversely, agents at inferior sites have lower suitability than they would have at IFD population levels. Agents may accept subordination to dominant agents within a site because this is better than the alternative of exit to an inferior site. Thus, with IDD, the suitability of agents can be unequal both across and within sites.

Some writers distinguish between *negative* and *positive* despotism [20]. In the former case, the early occupants at a site drive away newcomers. In the latter case, the early occupants extend concessions to newcomers and allow them to stay in subordinate roles so long as they provide labour services. Subordinates may find the concessions offered by despots attractive in comparison with the alternative of moving to the next best site. Weitzel & Codding discuss the costs and benefits of defending a site (negative despotism) and the costs to a despot of allowing people to settle at a site (positive despotism). They conclude their discussion of these issues with a comment that ‘understanding how these trade-offs articulate within an [ideal distribution model] to produce varied outcomes remains under-explored’ [20, p. 352].

Key empirical questions in this literature include the sequence in which individual sites within a region become occupied and how population is distributed across sites at a given point in time. Researchers often attempt to link these observations to underlying environmental factors that determine the qualities of the sites. Less attention is usually given to measures of inequality involving the fitness, nutrition or health of agents, either across or within sites.

To use the IFID framework as a theory about the origins of inequality, one would need to identify factors that could trigger the transition from an IFD to an IDD (either positive or negative). Empirical researchers concerned with this issue tend to highlight rising population density as a likely trigger. Although this is plausible, one then needs a theory where regional population is endogenous, by which we mean that regional population is causally determined by factors internal to the theory. One also needs data regarding the changes in suitability levels for individuals or groups. We will return to these matters in §6.

## 4. Insiders, outsiders, elites and commoners

Here we summarize our theory about the origins of inequality. The theory can be applied equally well to sedentary foragers and early farmers. The underlying mathematical framework is described at length elsewhere [4, ch. 6;^21^].

We preface this discussion with some methodological remarks. When archaeologists and anthropologists engage in formal modelling, they often use agent-based simulations. Economists also use simulation, but they have a long tradition of constructing models by adopting a few crisp assumptions and deriving results analytically. Our approach is in the latter tradition.

In this context, simplifying assumptions are vital, both to clarify the key causal pathways and for analytic tractability. To take some examples: in the discussion below, we assume that (i) it is costless to migrate among the sites within a region but impossible to move to a site outside the region; (ii) there is a local population threshold below which outsiders cannot be prevented from entering a site, and above which they can be excluded; (iii) insiders can deter entry through threats of violence that have no opportunity cost in terms of food production. We do not argue that these assumptions are accurate descriptions of reality, but we do argue that they provide a useful starting point for analysis, and that they have interesting implications.

Models of this kind can always be made more realistic by adding complexity to one's assumptions. However, several points should be borne in mind. First, models sometimes give surprising or counterintuitive results, and it is easier to understand the reasons for such results when the assumptions are kept simple. Second, it is rarely useful to start a formal analysis with a complex set of assumptions. It is much better to start simple and then explore the implications of changing one assumption at a time. Third, additional realism does not always lead to different implications. Introducing certain factors that were previously ignored could well reinforce the predictions from the original model. Fourth, simple models more often generate unambiguous predictions and thus are more open to empirical testing. Finally, if the predictions of a simple model line up well with known empirical patterns, further complication serves no real purpose. This is especially true if the simple model is also powerful in the sense that it provides a unified explanation for diverse phenomena. In this spirit, we describe the basics of the IOEC model.

Consider a region bounded by mountains, deserts, oceans or long distances from other inhabited regions. Migration between regions is negligible. A region has many production sites, where we use the term *site* in an economic sense to mean an area within which agents use labour and land to produce food. This differs from the archaeological meaning of a site as a location at which data are gathered. We use the terms *site* and *territory* interchangeably, but ‘territory’ has the connotation that the land area involved is relatively large. When a sizeable population resides permanently in a geographically compact area, we sometimes use the term *settlement* instead of site, but this concept does not play any distinct theoretical role.

Time periods are the length of one human generation (about 20 years). Within a period, the aggregate regional population is exogenous, but the agents can move among sites subject to constraints described below, so the local populations are endogenous. Each adult agent chooses a site, produces food, has children, and dies. Children become adults in the next period. Events in a single period are the *short run*. Events unfolding over multiple periods are the *long run*.

### (a) Short-run equilibrium

At each individual site, food output is determined by region-wide climate, resources and technology; the quality of the site; and the amount of labour used for food production (equal to the adult population at the site). Variations in site quality reflect variations in local geographical factors such as terrain, ecosystems, soil fertility and access to fresh water.

Labour exhibits diminishing returns owing to the fixed land input at a site (e.g. doubling labour input results in less than double the food output). The average product of labour is food output per unit of labour input. Diminishing returns imply that food per person falls when the local population rises. When the local population is low enough, anyone can enter the site and produce food there. In this case, the agents at the site share food equally and each receives the average product of labour.

There is a threshold for local population density (*d*) such that the existing occupants at a site, called the *insiders*, can block further entry. Groups of this size can reliably detect entry and carry out reprisals. For example, the insiders might cooperate to kill or drive away the outsiders. Threats of reprisal are credible and therefore potential entrants are deterred. Because deterrence succeeds, in equilibrium, there is no need to carry out the threatened actions, so the exclusion of outsiders has no opportunity cost in terms of food output. We assume insider groups of size *d* can overcome any coordination or free rider issues connected with the defence of a site and they share food equally among themselves. We think of *d* as involving the deterrence of individual unrelated outsiders. Cases involving groups of outsiders, or kinship links between insiders and outsiders, will be discussed briefly in §5.

Our framework assumes that potential entrants are intercepted at the boundary of a site and that the agents already occupying the site share its resources in an egalitarian way. We do not consider situations where the first household to arrive at a site can force the next household to accept land of lower quality at the same site. Although such situations do sometimes occur at sites in the archaeological sense, we ignore unequal access to resources within an insider group.

When choosing a production site in the short run, agents can freely enter any site having a local population below *d*. Such sites are called *open*. We define the *commons* to be the set of all open sites within a region. Agents cannot enter a site having a local population at or above *d* unless the insiders allow this. We call such sites *closed*.

The defining feature of a region is a low cost of travel among sites. This implies that all sites in the commons must have roughly the same average product in equilibrium because if any significant differences in average product existed, the agents would move from places with low average product to places with high average product. We use *w* to denote the equilibrium food income per person in the commons. Note, however, that equality of average products only holds for the subset of sites that are open. Sites that are closed, and therefore not in the commons, will have average products above *w*.

At closed sites, insiders may choose to hire some outsiders to work on their land. If they do, they need to offer workers a food income equal to *w* because this can always be obtained in the commons. We call this the *wage*. When insiders hire some outsiders, we say that the site is *stratified*, we refer to the insiders as an *elite*, and we refer to the hired workers as *commoners*.

Our analysis would be identical if instead of receiving a wage, the commoners paid a rent (again in food units) for the right to work on elite land and consumed their food output net of this rent. In our modelling, the elite at a given site are a cohesive group. We do not consider competition among factions within an elite, as might arise in large-scale societies.

Whether insiders hire outsiders depends on the marginal product of labour, defined to be the extra output that results from a small increase in labour input. When the marginal product of labour is less than *w*, the insiders at a closed site do not hire any outsiders because the latter add less to food output than their cost in wage payments. If the marginal product of labour is greater than *w*, the insiders will hire some outsiders, and employment of commoners expands until the marginal product of labour falls to the level *w*.

This framework yields the following results for short-run equilibrium.

(i) If the regional population is low enough, all sites are in the commons because the local population is below *d* at all sites. Better sites have higher populations.
(ii) If the regional population has an intermediate value, the best sites are closed but no sites are stratified. Lower-quality sites are in the commons. The closed sites have populations equal to the exclusion threshold *d*, while open sites have fewer people than this.
(iii) If the regional population is high enough, the best sites are stratified, sites of intermediate quality are closed but not stratified, and the worst sites stay open. Among stratified sites, better sites have more commoners, but all stratified sites have elites of size *d*.

### (b) Long-run equilibrium

The aggregate regional population adjusts through Malthusian dynamics. For simplicity, we assume parthenogenesis (all agents are female). Adults who have higher food incomes have more surviving children. There is a level of food income y* at which an adult has one surviving child.

In a long-run equilibrium, the regional population settles at a stationary size N* where the average product of labour for the region is y*. An improved climate or technology yields a higher regional population N* in long-run equilibrium but the average food income y* stays unchanged. Note that y* involves aggregate food output and aggregate population for the region. Individual closed sites will have a range of local average products depending on land quality.

When there is some inequality across or within sites, the agents with high food incomes produce more children than are needed to replace themselves demographically, while the agents with low food incomes produce fewer children than are needed for replacement. Hence, a stable class structure requires some downward mobility where a subset of the children of insider or elite parents become commoners in each period. The remaining children inherit insider or elite status at the sites of their parents. Commoner parents always have commoner children.

### (c) Implications

Region-wide productivity depends on region-wide climate and technology. If either of these factors improves, the short-run effect is a higher food income per person with an unchanged regional population. The long-run effect is a higher regional population with the same food per person as before the change in climate or technology. As with other Malthusian models, exogenous shocks (positive or negative) are absorbed in the long run through changes in population rather than changes in the average welfare of the individual agents.

Our theory combines endogenous population with endogenous property rights. A better climate or technical progress raises population densities in the long run for standard Malthusian reasons, but a higher regional population means that more sites achieve the minimum threshold for the exclusion of outsiders. Thus, the commons shrinks and the average quality of the sites in the commons declines. This depresses the food income of the agents in the commons. If regional productivity becomes sufficiently high, the falling wage induces stratification at the best sites.

Consider a region in which productivity is rising over time, for either natural or technical reasons. A crucial prediction of the model is that we should observe a sequence of stages within the region. At a low productivity level, implying low regional population, all sites are open, and equality prevails both within and across sites. At an intermediate productivity level, which gives an intermediate regional population, high-quality sites become closed, but no sites are stratified, so we should see continued equality within each site but inequality across sites, where agents at better closed sites are better off. We call this *insider–outsider* inequality. At a high productivity level and a resulting high regional population, high-quality sites become stratified, which leads to inequality both within and across sites, where agents who control better sites are again better off. We call this *elite–commoner* inequality.

Another implication of our story is that commoners become worse off in absolute terms as regional productivity increases. With more of the good sites closed, those who remain in the commons work on lower-quality land, food per person in the commons falls, and thus the wage paid to commoners at stratified sites also falls. Because the land rents enjoyed by insiders and elites are simultaneously rising owing to rising regional productivity, inequality rises both for the region as a whole and within the individual stratified sites.

## 5. Kinship and warfare

The parameter *d* in §4 is the critical mass of insiders required to deter individual unrelated outsiders. Insider or elite groups may sometimes be larger than this for two reasons.

### (a) Kinship

Insiders or elites may be willing to share land with outsiders related by biology or marriage, even if this leads to less food per person among the insiders. For example, kin from other locations might face adverse environmental conditions and seek refuge with relatives. One can think of land sharing in this instance as a form of insurance. Cases of this sort are less likely when settlements are large because members of large communities tend to marry endogamously, and thus have fewer kinship linkages with outsiders [22].

### (b) Warfare

Unrelated outsiders do not necessarily arrive one at a time. If insiders perceive a serious threat of attack from an organized group of outsiders, they may wish to expand the size of their own group. The economic trade-off is less food per person in peacetime versus a higher probability of winning in wartime. This leads to a theory of warfare over land among egalitarian groups [4, ch. 7;^23^]. In stratified societies, elites could grant elite status to victorious warriors who administer conquered lands [4, ch. 8].

The idea that kinship and warfare considerations affect settlement sizes is not uniquely ours. However, we want to point out that the IOEC framework can be extended to handle these considerations when appropriate (for details, see the citations in the two preceding paragraphs).

## 6. Comparisons of theories

This section compares our IOEC theory from §4 with HEHI from §2 and IFID from §3. While IOEC overlaps to some degree with these two alternative theories, in each case it provides explanations for phenomena that the other theories do not. On the other hand, each of the alternative theories focuses on some phenomena that are omitted from IOEC, at least in its current form. Thus, the three theories are best viewed as complements to one another.

### (a) Insider outsider elite commoner versus Holocene environment and household inheritance

Within the HEHI framework, the transition from the Pleistocene to the Holocene was important because it led to greater climate stability, as well as the widespread availability of dense and predictable resource patches. We agree about the importance of these factors but do not model them formally. In our framework, we treat climate improvement as a factor that enhances productivity, yielding more food output from fixed inputs of labour and land. In the long run, this productivity effect increases regional population, which influences property rights and inequality. In IOEC, improvements in climate and improvements in technology both tend to promote inequality because both are sources of long-run productivity growth.

HEHI also emphasizes that some sites are better than others, and that it matters whether agents find it feasible or desirable to defend the best sites. We agree with this point and model it explicitly. In IOEC, the prevalence of diminishing returns implies that insiders want to maintain control over good land whenever they can, and the feasibility of maintaining control depends on the density of insiders per unit of land. Insiders may either block outsiders from entering a site or allow them to enter as subordinates who supply labour but do not control land, depending on the quality of the site.

HEHI focuses on inequalities across individuals or households, while IOEC does not. Our theory is about inequality across groups: either insiders versus outsiders, or elites versus commoners. We are therefore concerned with the emergence of what we would call structural inequality, and we ignore individual variation within these classes. Conversely, HEHI ignores structural inequality among classes. Note, however, that we do not limit attention to stratified societies involving elites and commoners. We also explain the emergence of inequality across sites in situations where each individual site remains internally egalitarian. We expect that for any given region, insider–outsider inequality will precede stratification chronologically.

A central difference between HEHI and IOEC involves property rights and inheritance. HEHI tends to see property rights as being held by individuals or households, and it is therefore concerned with the transfer of asset ownership from parents to children within a household. We tend to see property rights over land as being established and maintained by groups, and in IOEC, land is collectively rather than individually owned. Specifically, we think of land as being held by corporate descent groups, with individuals inheriting membership in such groups rather than directly inheriting private property rights over land parcels. In some applications, however, we do consider the possibility that individual members of an elite could have private rights to land and hire commoners to work on their individual estates.

HEHI and IOEC also emphasize different causal channels. IOEC focuses on the long-run effect of productivity-related variables such as climate, geography and technology on aggregate regional population. We model how the resulting regional population will be distributed across sites in the short run, which enables us to endogenize the property rights prevailing at each site in the region. We can then explain how improvements in climate or technology give rise to greater inequality through the mediating effects of population and property rights.

HEHI focuses on causal channels running from technology (hunter–gatherer, horticulture, agriculture and pastoralism) to the relative importance of different wealth types (embodied, material and relational), and from the heritability of each wealth type to the degree of individual inequality in a society using a specific technology. Our theory does not rely on a technological classification system of this kind, and it applies equally well to foragers and farmers. On the other hand, we have only a single asset that can be inherited (land).

For reasons of data availability, HEHI authors tend to study hunter–gatherer societies that are located toward the egalitarian side of the foraging spectrum [11,24,25]. Sedentary hunter–gatherers with high levels of inequality, up to and including class stratification, are known both archaeologically and historically. Classic examples include societies along the northwest coast of North America [26,27]. Other examples include the Calusa and the Chumash. Such societies were often based on concentrated aquatic resources. HEHI writers note the importance of these societies, but they are no longer extant and cannot be used to estimate the relative importance or heritability of wealth types. Even so, for our purposes, it is best to disaggregate mobile and sedentary hunter–gatherers, because the former rarely have persistent institutionalized inequality while the latter sometimes do. Given suitable archaeological data, one can use the IOEC framework to explain the varying degrees of inequality exhibited by prehistoric sedentary foragers.

Some writers in the HEHI literature stress the difference between labour-limited and land-limited farming [10]. This is one aspect of the definitional distinction between horticulture and agriculture drawn by Borgerhoff Mulder *et al*. [11], who find that horticulture (labour-limited) has a relatively low level of inequality, while agriculture (land-limited) exhibits a much higher level of inequality. From an IOEC perspective, this correlation can be explained by the fact that Malthusian population growth with fixed land resources will shift a region in a more land-limited direction over time, and this trend will coincide with rising inequality.

### (b) Insider outsider elite commoner versus ideal free ideal despotic

Similarities between our theory and IFID are easy to see. To take one example, our agents maximize food consumption, but this is linked to an adult's surviving offspring, so in effect our agents maximize fitness. The latter is often called 'suitability' in the IFID literature. Thus, IOEC and IFID adopt parallel assumptions about agent motivation.

Another similarity involves the distinction between open and closed sites. When all sites have local population densities below our threshold ‘*d*’, all sites are open. In this case, our IOEC concept of short-run equilibrium is identical to the concept of an IFD used by IFID. Once a site reaches this population threshold, further entry is blocked. This generates an IDD among closed sites as in IFID, where insiders who control better sites have more food *per capita*. Specifically, our concept of insider–outsider inequality corresponds to the idea of negative despotism (exclusion of newcomers) in IFID.

We also identify conditions under which commoners will accept subordinate positions in relation to an elite who control access to a site. The concept of elite–commoner inequality in our theory corresponds to the concept of positive despotism (concessions to newcomers) in IFID. In some cases, our model of stratification might be interpreted as a House society where the elite at a site are linked by kinship while the commoners are unrelated or more distantly related [28]. It might also be interpreted as a system of patron–client relationships [29, pp. 325–327;^30^]).

The IFID literature frequently focuses on the question of whether an individual site will be occupied, and it links such occupation patterns to rankings of site qualities, where population growth tends to bring lower-quality sites into use. By contrast, our IOEC models typically have the feature that all sites are occupied, but those with very low quality have very few occupants. Hence, in its current form, the IOEC model is not well suited to the task of explaining whether a specific site is occupied. In principle, however, the IOEC model could be modified by adopting a different specification for the food technology, where the number of agents at low-quality sites is zero in equilibrium. With this modification, we would get the usual IFID prediction that as the regional population grows, lower-quality sites will successively come into use.

Another distinction between the two frameworks is that IFID frequently emphasizes the role of Allee effects, in which suitability increases as the initial agents arrive at a site, reaches a maximum and then decreases as more agents arrive. The initial interval with increasing returns could arise from productivity gains associated with teamwork or a division of labour, where these gains decline or are exhausted at sufficiently large scales and diminishing returns to labour due to a fixed land input eventually dominate.

In our current IOEC models, we use a simpler production technology, where diminishing returns to labour prevail regardless of the number of agents at a site. However, one could modify our technological assumptions to include an initial interval in which the average product of labour rises, with a falling average product thereafter. Such models are often used in economics and do not pose any problem in principle, although this would complicate the formal analysis. The main effect would be to create discontinuities where individual sites could jump from zero to positive populations in response to changes in aggregate regional population. We would not observe sites located on the rising part of the average product curve because such equilibria would be unstable.

The central issue for IFID in the present context involves the causal factors that trigger a shift from an IFD with equality to an IDD with inequality. Empirical researchers using the IFID framework generally cite population growth as a key factor (see the discussion of the Channel Islands, Neolithic Europe, Polynesia and the Maya in §7). We agree about the causal importance of population, and our parameter *d* for the density of insiders at a site constitutes the dividing line between ideal free and ideal despotic distributions.

The long-run Malthusian component of the IOEC model provides a causal link running from climate and technology to aggregate regional population. The short-run component of the model determines the regional population levels that will be associated with (a) open access, (b) insider–outsider inequality and (c) elite–commoner inequality. Our theory generates predictions about when a shift from (a) to (b) will occur, which can be interpreted in the IFID framework as a transition from an IFD to a distribution with negative despotism. IOEC also generates predictions about when a shift from (b) to (c) will occur, which can be interpreted in the IFID framework as a transition from negative to positive despotism. In the first case, sites are internally egalitarian, and in the second case, they are internally stratified.

An important IOEC prediction is that open, closed and stratified sites can coexist in the same region, where low-quality sites are open, intermediate sites are closed but unstratified, and high-quality sites are both closed and stratified. To put the same idea into IFID terminology, the region can exhibit an IFD among one subset of sites, negative despotism among another subset, and positive despotism among a third subset. IOEC also yields predictions about when a site of given quality will transition from one property rights regime to another.

One advantage of our theory in comparison with IFID is that it gives clear mathematical predictions about not only the distribution of population across sites, but also the distribution of food income across agents. Accordingly, IOEC provides a more direct foundation for the study of inequality. It also addresses various economic linkages among productivity, inequality and inefficiency. We show that improvements in climate or technology, while raising productivity, simultaneously impoverish commoners. The reason is that property rights are endogenous and the commons shrinks as productivity increases. As this process unfolds, the economy becomes inefficient in the sense that aggregate regional food output falls below its theoretical maximum (it would be possible to raise total output by transferring labour from poor sites in the commons to good sites that are stratified). These effects are easier to see with IOEC than with HEHI or IFID.

## 7. Regional cases

This section surveys some evidence bearing on our IOEC theory from §4. These examples are meant only to illustrate how our theory could be applied to empirical cases. For brevity, we devote one paragraph to each case and omit many details of interest to experts.

### (a) Western North America

Using a sample of 157 foraging societies, Codding *et al*. [31] find that larger local groups are more likely to claim ownership rights to resource locations. This relationship is strong for foragers focused on terrestrial plants and aquatic food resources, but not those focused on hunting. Their ownership variable roughly corresponds to our concepts of open access, closed access and stratification. The results are consistent with our prediction that, other things equal, local groups having larger sizes are more likely to reach the critical mass needed to exclude outsiders from a site. Related work by Smith & Codding [32] shows that hierarchy is associated with control over concentrated aquatic resources. From our standpoint, this shows that such resources can support elite–commoner inequality in sedentary foraging societies.

### (b) The Channel Islands

Jazwa *et al*. [33] study the transition from IFD to IDD on Santa Rosa Island, which they associate with the emergence of chiefdoms around 1300 BP. However, their fig. 6 [33, p. 51] shows rising rates of cribra orbitalia and periosteal lesions over time, with declining stature for men, during 2200–1000 BP. This suggests gradually deteriorating standards of living for much of the population well before visible stratification. We propose that technical innovations (single-piece fishhooks by 2500 BP and plank canoes by 1500 BP) supported rising regional populations, leading to sequential closure of higher-quality sites throughout 2200–1300 BP. Such site closures would have caused worsening poverty among outsiders (IO inequality) well before overt stratification emerged around 1300 BP or later (EC inequality). On a larger geographical scale, insider–outsider inequality can be inferred from differences across islands in the rate of cribra orbitalia [4, p. 267;^34^]. This marker for anaemia was more frequent on islands with poorer resources, even though movement among islands would not have been physically difficult.

### (c) Neolithic Europe

Shennan [17,18,35] describes the emergence of inequality for the first farmers to settle in central Europe. Initial settlements seemed to be relatively egalitarian with broad individual mobility. As population increased, favourable locations were filled in, with early arrivers maintaining control over the locations settled first. Cemeteries became common, suggesting claims to ancestral territory. Evidence for an insider–outsider stage includes variation across sites in house sizes, tools and domestic animal bones. Increasingly, it was mainly women who moved, suggesting the formation of patrilineal corporate groups. This is consistent with our expectation that higher local populations lead to inherited membership for insider groups and the exclusion of outsiders. Eventually populations became high enough to yield stratification at the best sites, as indicated by differences in house sizes and grave goods within settlements.

### (d) Southeast Asia

Fochesato *et al*. [36] examine the trajectory of inequality in the Upper Mun Valley in Thailand from the arrival of Neolithic rice farmers around 2000 BC to the formation of early states around 500 AD. They use grave goods to compute Gini coefficients for several sites in the region at various points in time. The Ginis for all Neolithic sites are relatively low while those for Bronze Age sites are higher (except one outlier at the end of this period) and similarly for Iron Age sites. At one key site, there is no evidence for inequality in the Neolithic. The authors highlight three surges in inequality within sites. The first two (at one Neolithic site *ca* 1800 BC and one Bronze Age site during 1100–800 BC) seem related to trade monopolization, were associated with clear elite–commoner divisions, and were temporary. The third (at multiple late Iron Age sites) was connected to aridity, a shift from rainfed dryland farming to irrigated wet fields, and increasing regional population. The higher inequality was permanent and early states developed relatively rapidly. The authors only discuss inequality within sites rather than across sites, so we do not know if or when there was any transition from open to closed sites during the 1000 years of Neolithic farming. However, technological innovation, population growth and the increasingly sharp stratification at late Iron Age sites are consistent with our theory in §4.

### (e) Polynesia

At historic contact, island chains in Polynesia showed strong cross-sectional correlations among the productivity of natural resources, population density and the degree of inequality [4, pp. 264–265]. This pattern conforms to our expectations based on the theory in §4. Archaeological evidence indicates that the initial settlement of Fiji and West Polynesia around 3000 BP was followed by a lengthy period of population growth, as we expect from Malthusian adjustment toward a long-run equilibrium. Kennett & Winterhalder [15, p. 92] remark that in this context, ‘competition for land would have been an important factor in the emergence of social hierarchies, but direct archaeological evidence for these hierarchies is meager until about 1000 BP’. Nevertheless, there is evidence for corporate group formation and inter-group conflict in Fiji and West Polynesia during 1500–1000 BP, such as hilltop settlements and fortifications. We can infer from the clear threat of warfare that insider–outsider inequality existed in this period [4, ch. 7;^23^] and was followed by elite–commoner inequality, starting around 1000 BP.

### (f) The Maya

Prufer *et al*. [37] examine the transition from an IFD to an IDD for the Classic Period Maya. The area of Uxbenka initially had a small population, perhaps around 40 people. As one expects from Malthusian dynamics under favourable environmental conditions, population growth followed. The authors suggest that a core area was settled first, followed by a periphery, with open access keeping the agents equally well off. However, over time the early sites, which were also the larger sites, developed lineal kinship organization, and agents in the periphery had lower status based on descent. Prufer *et al*. believe individuals in the periphery did not migrate to take advantage of better opportunities in the core because kinship led to locational stickiness (people did not want to leave their own close kin or accept distant kin from other locations). The authors propose that this was sufficient for a transition from IFD to IDD (or in our terms, from open to closed sites). There is no evidence of stratification during 300 BC to 200 AD, but by around 200 AD public works, public architecture, and landscape modification indicate stratification. At this point, the population had risen to about 500. Commoners appear to have had reasonably good outside options (outlying areas had arable land and reliable water supplies), but the authors suggest that peripheral areas were impoverished relative to the elite core. During 400–800 AD, the elite made fewer concessions to non-elites, which we interpret as falling wages for commoners owing to a rising regional population and the diminishing quantity and quality of sites in the commons.

## 8. Conclusion

The IOEC theory we have developed in this paper and in Dow & Reed [4, ch. 6]) has the following causal structure.

(a) Improvements in climate and/or technology raise regional productivity and lead to long-run aggregate population growth through Malthusian dynamics.
(b) Regional population growth leads to higher local population densities at individual sites, causing an initial closure of the best sites (insider–outsider inequality) and the subsequent stratification of the best sites (elite–commoner inequality).
(c) Over time, the extension of insider and elite property rights to lower-quality sites leads to a contraction of the commons, with the lowest-quality sites remaining open.
(d) Thus, productivity growth due to improved climate or technology yields greater regional inequality, more inequality at individual stratified sites and worsening absolute poverty for commoners.

We provide formal economic reasoning for each stage in this process.

Related theoretical frameworks follow similar trajectories but leave out some elements of this story. The HEHI approach in §2 goes from the Holocene to economic defensibility of good sites, to the increased importance of material assets in agriculture and pastoralism, and then to greater inequality among households or individuals. However, it ignores group property rights and provides no causal explanation for insider–outsider inequality or elite–commoner inequality. The IFID approach in §3 has a similar property rights sequence to IOEC, going from open sites to closed sites to stratified sites, but it is less explicit about the reasons for these transitions and their consequences for inequality.

The core of our theory involves local population density and the critical mass of insiders per unit of land area that would be sufficient to exclude outsiders. The main way in which this critical mass can be reached is through Malthusian population growth at the regional level. But other mechanisms could lead to local population growth at some sites. For example, people may respond to negative environmental shocks by migrating to refuge sites that are buffered from the shock, or they may respond to threats of warfare by migrating to easily defended sites, or they may migrate toward newly profitable trade routes. Any of these processes could yield local IO or EC inequality even without population growth at the regional level. Cultural factors such as shifts in religious beliefs might also make individual sites more attractive, with migration again generating population agglomeration and greater local inequality.

We understand that non-economists may be uncomfortable with some of the simplifying assumptions used in our IOEC theory: for instance, the binary distinction we draw between open and closed sites, and our use of a particular population threshold to switch from one to the other. However, other theories draw similar distinctions between sites that are economically defensible and those that are not, or between ideal free and ideal despotic distributions. We invite sceptics to construct their own models using their preferred assumptions. But in the meantime, IOEC has several advantages. It is explicit about causality, it offers a unified explanation for a wide range of phenomena, and it has a rich assortment of empirical implications.

HEHI, IFID and IOEC share a focus on changing environmental conditions and control over valuable resource locations as important drivers of early inequality. Other theories put the focus elsewhere. For example, some archaeologists maintain that most or all societies include aggrandizers who try to promote their own welfare at the possible expense of others, and that, in periods of stress (environmental, technological, demographic or the like), dominant individuals seize greater control over resources or shape social institutions in ways favourable to stratification [16,38]. Relatedly, some writers emphasize efforts by aggrandizers to gain direct control over the labour time of others rather than control over physical assets such as land [39,40]. Others emphasize the ability of individuals or groups to manipulate social norms or cultural beliefs in ways that enhance their own privileges [41]. We do not dismiss theoretical stories of this kind, but they are less explicit about causal mechanisms than the approaches we discussed earlier, and they seem more difficult to test.

None of the cases in §7 provides comprehensive support for the IOEC theory in §4, but we hope these cases suggest the general plausibility of our story. One lesson we derive from these cases is that empirical researchers often define the starting point for inequality in a region using the initial appearance of stratification (e.g. evidence for chiefdoms). We think this tends to understate the antiquity of inequality by neglecting a potentially prolonged insider–outsider stage during which technological innovation and population growth were leading to the closure of good sites, resulting in greater inequality across but not within sites.

Better tests of the IOEC model would require a panel of individual sites within a region, ranked according to their natural productivity and observed at various points in time. We would want skeletal evidence on nutrition or health that could be used to compute means and variances for individual welfare at each site and date. Contextual information on the dynamics of regional climate, technology and population would be valuable. In a perfect world, it would help to have information for each site and date on local population size, the prevailing property rights system (open, closed or stratified), and inheritance rules governing individual membership in insider or elite groups. Such datasets would permit a more systematic evaluation of the IOEC framework.

## Data Availability

The verbal discussion in our paper relies upon results obtained from a formal mathematical model that has been published in the *Journal of Political Economy* [21] and in our book ‘*Economic prehistory: six transitions that shaped the world*’ (Cambridge University Press, 2022) [4]. Full citations to both sources are given in the reference list for the present paper.
